# Clinical Evaluation of Autologous PRP (Platelet-Rich Plasma) in the Treatment of Periodontitis in Small-Breed Dogs

**DOI:** 10.3390/ani15243581

**Published:** 2025-12-12

**Authors:** Dmitrij Kvitka, Martinas Jankauskas, Matas Klupšas, Aistė Gradeckienė, Dalia Juodžentė, Greta Rudenkovaitė

**Affiliations:** Veterinary Academy, Lithuanian University of Health Sciences, 44307 Kaunas, Lithuania; martinas.jankauskas@lsmu.lt (M.J.); matas.klupsas@lsmu.lt (M.K.); aiste.gradeckiene@lsmu.lt (A.G.); dalia.juodzente@lsmu.lt (D.J.); greta.rudenkovaite@lsmu.lt (G.R.)

**Keywords:** regenerative therapy, periodontitis, periodontal regeneration, platelet-rich plasma (PRP), PRP injections, small-breed dogs, veterinary dentistry

## Abstract

Periodontal disease is a progressive inflammatory condition frequently diagnosed in dogs, particularly in small breeds such as Yorkshire Terriers, Toy Terriers, Spitz, Toy Poodles and other breeds predisposed to rapid plaque and tartar accumulation. As the field of regenerative medicine becomes more popular, more and more attention is being paid to substances that promote tissue regeneration, one of which is platelet-rich plasma (PRP). PRP is an autologous blood-derived product rich in growth factors that stimulate tissue regeneration and modulate inflammation. This study aimed to evaluate the clinical effectiveness of PRP injections without additional activating agents in the management of stage 2–3 periodontitis in small-breed dogs. Forty-two adult dogs (Yorkshire Terriers, Toy Terriers, Pomeranians, Toy Poodles, and Havanese) were enrolled and divided into two groups: PRP (*n* = 30) and control (*n* = 12). Following standard dental prophylaxis, the PRP group received gingival, submucosal, and periodontal pocket injections of PRP (0.1 mL per site). Periodontitis stage, gingival index, periodontal pocket depth, and horizontal bone loss were evaluated at baseline and 30 days post-treatment. PRP therapy significantly improved all evaluated parameters (*p* < 0.05). The gingival index decreased threefold, periodontal pocket depth was reduced twofold, and horizontal bone loss decreased by more than twofold compared with baseline and controls. No adverse reactions, discomfort, or postoperative complications were observed. The administration of non-activated PRP as an adjunct to dental cleaning significantly enhances soft and hard tissue regeneration in small-breed dogs with stage 2–3 periodontitis. PRP therapy represents a safe, minimally invasive, and effective regenerative approach for improving periodontal health in routine veterinary dentistry.

## Simple Summary

Periodontal disease is among the most common oral health problems in dogs, affecting up to 80% of animals by two years of age. Small-breed dogs such as Yorkshire Terriers, Toy Terriers, Pomeranians, and Toy Poodles are particularly susceptible due to their genetic predisposition to rapid plaque accumulation and early-onset periodontitis. Platelet-rich plasma (PRP), a regenerative therapy derived from autologous blood, has shown promising results in promoting tissue healing and reducing inflammation. This study investigated the clinical effectiveness of PRP injections without additional activating agents in the routine treatment of stage 2–3 periodontitis in small-breed dogs. Forty-two dogs were divided into PRP and control groups. After 30 days, the PRP-treated dogs exhibited significant reductions in gingival inflammation, periodontal pocket depth, and horizontal bone loss compared with controls. No adverse effects were observed. These results demonstrate that PRP can serve as a safe, minimally invasive, and effective adjunctive therapy for improving periodontal health and promoting tissue regeneration in small-breed dogs.

## 1. Introduction

Oral cavity diseases represent the most common pathological conditions diagnosed in dogs of various ages during routine clinical examinations, with periodontal disease being the most frequently encountered [1,2,3]. It is estimated that up to 80% of dogs as young as two years old suffer from some form of periodontal disease [1]. This condition is defined as a chronic inflammatory state of the tissues surrounding the tooth, induced by bacteria located beneath the dental plaque and the host’s immune response [3].

Periodontitis specifically refers to the inflammatory destruction of deeper supporting structures, such as the periodontal ligament and alveolar bone, caused by microbial activity [3,4]. Black-pigmented anaerobic bacteria possess several virulence factors, including direct epithelial invasion, production of collagenases and proteases, endotoxin release, inhibition of neutrophil function, and stimulation of host pro-inflammatory cytokines [5]. These mechanisms allow bacteria to evade immune defenses, promote tissue destruction, and contribute to the progression of periodontal disease [6,7]. Severe cases are characterized by deepened periodontal pockets, gingival recession, increased tooth mobility, furcation exposure, and eventual tooth loss. Gingivitis represents the earliest stage of periodontal disease, manifested by clinical signs such as gingival erythema and edema [8].

Gingivitis typically begins at the gingival margin, where tissues become reddened and rounded; with progression, spontaneous bleeding and ulceration may occur [3,8]. If the underlying cause is not eliminated and inflammation persists, gingival detachment from the tooth occurs, leading to the formation of periodontal pockets [3,9].

Standard periodontal therapy includes plaque removal, tooth polishing, use of prophylactic measures against plaque and calculus accumulation, and at-home dental care [9,10]. In small-breed dogs, such as Yorkshire Terriers, Toy Terriers, Spitz, Toy Poodles, and other breeds genetically predisposed to rapid plaque/calculus accumulation and periodontitis, dental cleaning must be performed relatively frequently [11]. However, owners often express concern regarding the risks and potential complications of general anesthesia [2,12].

With advances in veterinary medicine and the increasing interest in regenerative medicine, more attention has been devoted to substances that stimulate natural tissue regeneration. One such therapy is platelet-rich plasma (PRP). PRP belongs to regenerative medicine—a broad field aimed at restoring or replacing damaged tissues and structures [13]. It is thought to modulate immune responses, promote healing, suppress inflammation, and reduce pain [13,14]. Due to centrifugation, PRP contains platelet concentrations three to five times higher than physiological levels in blood [13,15]. PRP was first introduced into clinical practice in 1986 during cardiovascular surgery, where platelet transfusion reduced donor blood requirements [16]. Since then, PRP applications have expanded to dentistry, maxillofacial surgery, and bone regeneration and implantation [13,17].

PRP’s therapeutic effects are mediated by bioactive platelet-derived factors, including platelet-derived growth factor (PDGF), fibroblast growth factor (FGF), vascular endothelial growth factor (VEGF), epidermal growth factor (EGF), among others [10,18]. Upon injection, platelets activate and release mediators that act on surrounding cells. PDGF and VEGF stimulate angiogenesis, endothelial cell proliferation, and the recruitment of leukocytes and fibroblasts to the injury site [19,20]. These processes enhance capillary formation and nutrient delivery [18]. FGF promotes fibroblast activation and collagen/fibronectin synthesis, while EGF stimulates epithelial proliferation and tissue integrity restoration [20]. Platelet-derived mediators, including ligands and prostaglandin E_2_, also contribute to anti-inflammatory responses by upregulating anti-inflammatory cytokines such as interleukin-10 [21]. Moreover, PRP exhibits bactericidal and bacteriostatic effects via antimicrobial peptides, reducing infection risks at lesion sites [20,22].

Several PRP derivatives exist, differing in composition, clinical application, and preparation. Platelet-rich fibrin (PRF), a more recent variant, excludes anticoagulants, allowing a fibrin matrix to release growth factors gradually [23]. A commercial system, PRGF (plasma rich in growth factors), contains a higher concentration of growth factors and is often used for aesthetic purposes [24].

A recent study demonstrated that calcium chloride-activated PRP injections into subgingival tissues, combined with dental hygiene, successfully treated grade 2 and 3 canine periodontitis. After 56 days, periodontal pocket depth, vertical bone loss, gingival index, and disease stage were significantly reduced [9]. However, the study involved a small sample size and was performed on individual teeth. PRP is prepared from autologous blood, making it a rapid, safe, minimally invasive, and resource-efficient process [4]. Due to its regenerative and anti-inflammatory properties, PRP may serve as an alternative or adjunctive therapy in the routine management of canine periodontal disease [4,9].

Given the scarcity of data and scientific evidence regarding PRP’s routine application in dogs, the aim of this study was to evaluate whether PRP therapy, applied without additional activating agents, produces any significant improvement in periodontal parameters or treatment outcomes compared with standard periodontal therapy alone.

## 2. Materials and Methods

### 2.1. Animals

This study, designed to evaluate the effect of routine PRP application, included 42 adult small-breed dogs (7 male and 5 female Yorkshire Terriers, 4 male and 2 female Pomeranians, 2 male and 7 female Toy Poodles, 5 male and 3 female Toy Terriers, and 4 male and 3 female Havanese); both sexes; 3–4 years of age; body weight 1.8–5.9 kg; feed only dry food; did not received home dental care) presented for routine dental prophylaxis.

This was a prospective, non-randomized controlled study. Dogs were consecutively enrolled based on the inclusion and exclusion criteria (Table 1). Group allocation was determined by owner consent: dogs whose owners agreed to PRP treatment were assigned to the PRP group (*n* = 30) and received PRP injections into the gingival subgingival tissues and periodontal pockets following dental prophylaxis. Dogs whose owners declined PRP formed the control group (*n* = 12) and underwent dental prophylaxis alone. The inclusion and exclusion criteria for all participants are summarized in Table 1.

Stage 1 lesions were excluded because clinical changes are minimal and reversible; after plaque and calculus removal, the gingiva typically returns to a physiologically normal state. Stage 4 lesions were excluded because all supporting tooth structures are severely compromised, with potential tooth mobility and abscess formation.

Ethical approval for this study was granted by the Veterinary Section of the Bioethics Centre at the Lithuanian University of Health Sciences (No. 2023-BEC2-430). All procedures were performed with informed owner consent and in accordance with animal welfare regulations.

### 2.2. Treatment

Prior to dental prophylaxis, each animal underwent a clinical examination including assessment of heart rate, respiratory rate, capillary refill time, superficial lymph node size, femoral pulse, and body temperature. A venous catheter was placed in either v. cephalica or v. saphena, and blood samples were collected for hematological analysis using the IDEXX ProCyte Dx hematology analyzer (IDEXX, Hoofddorp, The Netherlands).

Premedication consisted of maropitant (1 mg/kg; Pfizer, Sandwich, UK). General anesthesia was standardized across all patients, induced with dexmedetomidine hydrochloride (3 µg/kg IV; CP-Pharma, Burgdorf, Germany) and methadone hydrochloride (0.3 mg/kg IV; Richter Pharma, Wels, Austria), followed by propofol (2–4 mg/kg IV; Fresenius Kabi, Bad Homburg vor der Höhe, Germany). Animals were intubated with an endotracheal tube, and anesthesia was maintained with propofol infusion (0.5–2 mg/kg). Throughout and after the procedure, body temperature was maintained using a BUSTER ICU warming pad (Kruuse, Langeskov, Denmark), and vital signs were continuously monitored with a Mindray uMEC12 Vet monitor (Mindray, Shenzhen, China).

**Figure 1:**
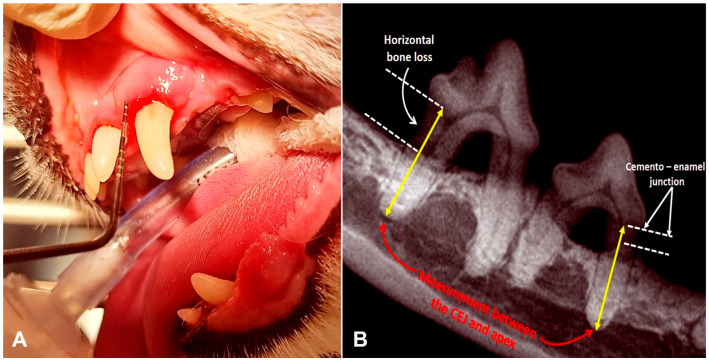
Periodontal pocket and horizontal bone loss measurements. (**A**) Periodontal pocket measurement with a graduated periodontal probe. (**B**) Measurement of horizontal bone loss in the radiograph of teeth 406 and 407.

Dental plaque and calculus were removed using an ultrasonic scaler (iM3 SP6, iM3, Duleek, Ireland). Periodontal pockets were probed and measured with a periodontal probe in millimeters (Figure 1A), and the stage of periodontitis (0–4) and gingival index (0–3) were recorded (Table 2). Intraoral radiographs of affected teeth were obtained using CS 2200 (Carestream, Atlanta, GA, USA), and horizontal bone loss was assessed by measuring the distance from the cemento–enamel junction to the alveolar crest (Figure 1B). Following scaling and radiography, all teeth were polished with prophy paste (iM3, Duleek, Ireland). A single subcutaneous dose of robenacoxib (2 mg/kg; Elanco GmbH, Cuxhaven, Germany) was administered post-procedure as nonsteroidal anti-inflammatory therapy.

### 2.3. PRP Preparation and Injection

**Figure 2:**
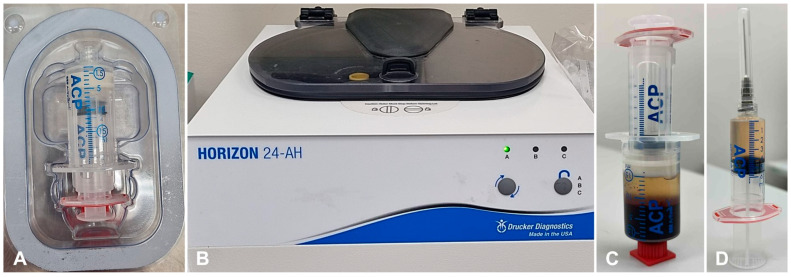
PRP preparation steps. (**A**) Arthrex ACP dual syringe system for blood collection. (**B**) A centrifuge that is used to centrifuge blood in double syringes. (**C**) Centrifuged blood, during which platelet-rich plasma is separated from other blood components in a double syringe. (**D**) The platelet-rich plasma is collected in a second syringe and the product is ready for use.

During general anesthesia, at the end of the dental cleaning procedure, 15 mL of blood for PRP preparation was collected from the jugular vein using the Arthrex ACP (Autologous Conditioned Plasma) double-syringe system (Arthrex GmbH, Munich, Germany) (Figure 2A) connected to a 21-gauge butterfly needle. The Arthrex ACP double-syringe system is designed to facilitate the safe and rapid preparation of autologous PRP from a small blood sample at the patient’s point of care. The Arthrex ACP syringes are not pre-filled with anticoagulant. When using this system, and if the PRP is injected within 30 min of blood withdrawal, the use of anticoagulant is not required.

The collected blood was centrifuged using the ARTHREX HORIZON 24-AH centrifuge (Arthrex GmbH, Munich, Germany) at 1500 rpm (402 g) for 5 min (Figure 2B). After centrifugation (Figure 2C), in order to transfer PRP from the larger outer syringe into the small inner syringe, we slowly pushed down on the outer syringe while slowly pulling up the plunger of the small inner syringe. PRP was separated from the remaining blood components (erythrocytes and leukocytes) (Figure 2D). The average time from blood collection to PRP preparation and injection was 10–15 min.

**Figure 3:**
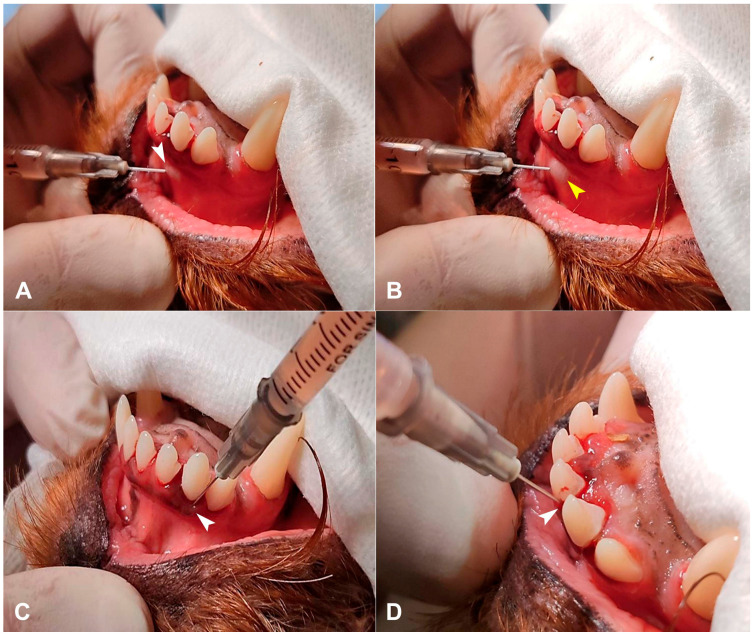
PRP injection sites. (**A**) The injection of PRP into submucosal tissue of 102 tooth. (**B**) After the injection of PRP into submucosal tissue of 102 tooth a small lump and mild swelling in the submucosal area was noted (marked with a yellow arrowhead). (**C**) PRP injection beneath the gingiva. (**D**) PRP injection into periodontal pockets at ligament attachment sites. White arrowheads mark the injection sites.

After dental cleaning and polishing, PRP was injected into the submucosal tissue, beneath the gingiva, and into periodontal pockets at ligament attachment sites, using a 1 mL insulin syringe connected to a 30-gauge needle, administering 0.1 mL per site (Figure 3).

### 2.4. Follow-Up Evaluation

No teeth were extracted in either the control or PRP groups; therefore, no additional NSAIDs or antibiotics were prescribed at home Owners were instructed not to perform any supplementary dental care at home for 30 days.

**Figure 4:**
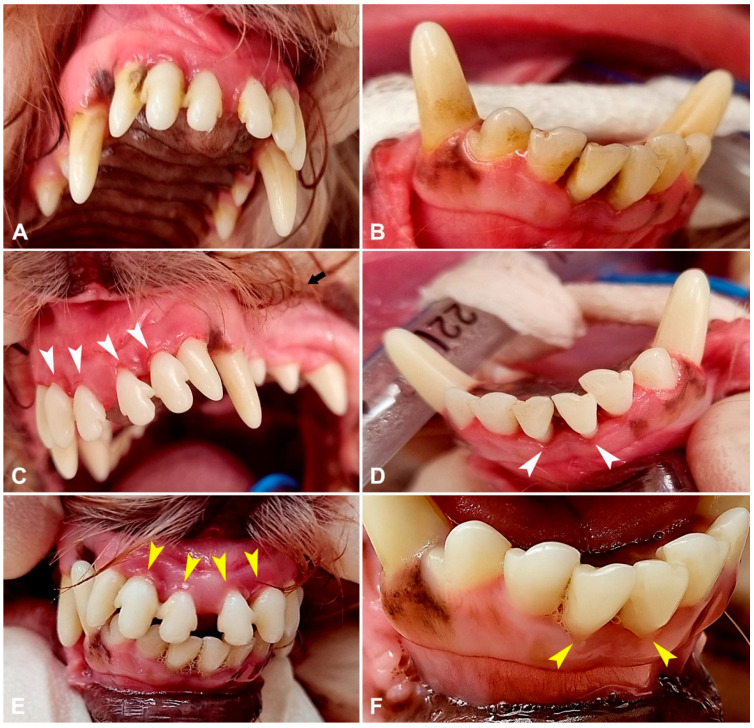
Photos illustrating the treatment processes of periodontitis. Figures (**A**,**B**) indicates upper and lower jaw teeth before the dental cleaning. Figures (**C**,**D**) indicates upper and lower jaw teeth after the dental cleaning. White arrowheads indicate areas of macroscopically visible gum lesion and recession caused by periodontitis after teeth cleaning. Figures (**E**,**F**) indicates upper and lower jaw teeth after PRP injections at follow-up 30 days post-procedure. Yellow arrowheads indicate macroscopically visible improvements in gum condition.

**Figure 5:**
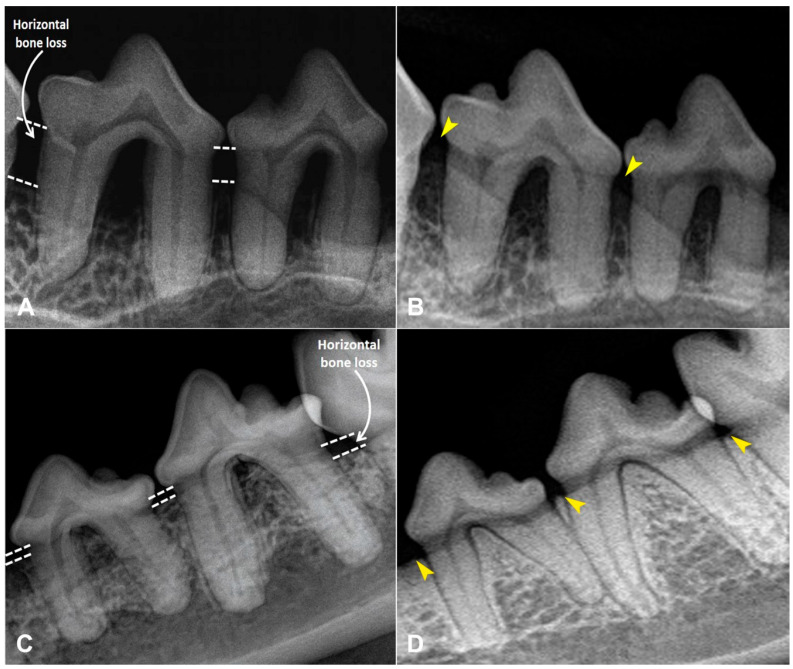
Radiographic images illustrating the positive changes in bone levels after the PRP treatment. Figure (**A**) indicates horizontal bone loss around teeth 106 and 107 prior to PRP treatment. Figure (**B**) indicates notable enhancement in jawbone condition at follow-up 30 days post-procedure. Figure (**C**) indicates horizontal bone loss around teeth 307 and 308 prior to PRP treatment. Figure (**D**) indicates evident improvement in jawbone condition at follow-up 30 days post-procedure. Yellow arrowheads highlight areas of visible bone regeneration.

At follow-up (30 days post-procedure), animals underwent general anesthesia as previously described. Periodontal pocket depth was re-measured, the stage of periodontitis and gingival index were reassessed, repeat dental radiographs were performed, horizontal bone loss was re-measured and the effectiveness of treatment was assessed (Figure 4 and Figure 5).

### 2.5. Study Selection

**Figure 6:**
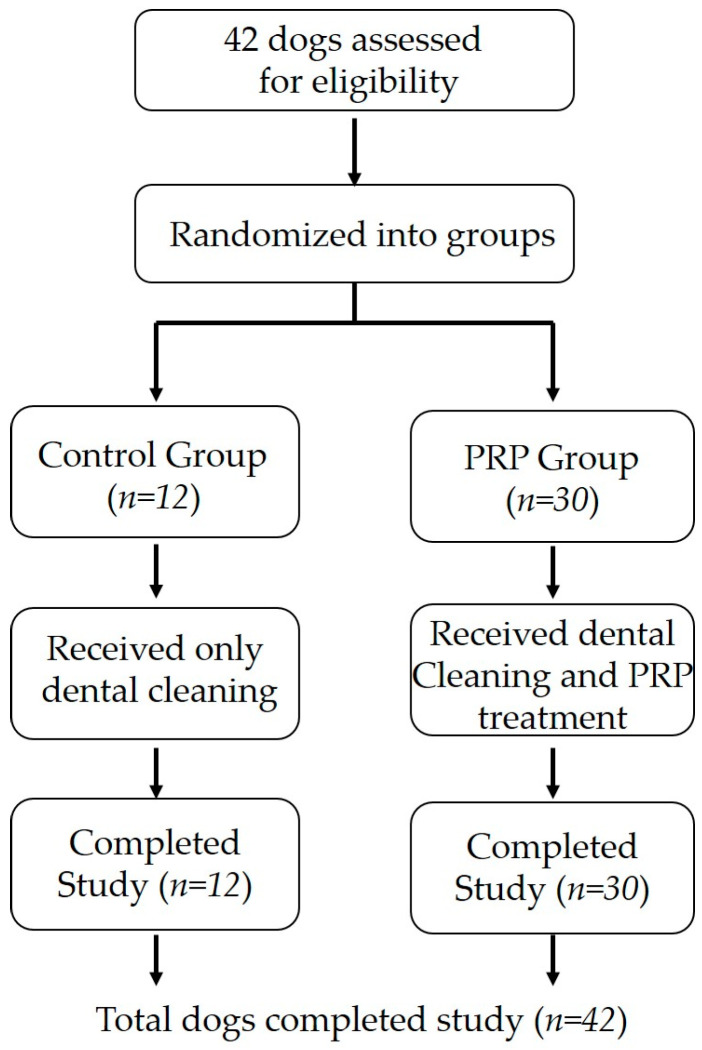
Study flow diagram.

In this study, no dogs were excluded and no dropouts occurred at any stage of the procedure or during follow-up evaluation. Flow diagram illustrating enrollment, allocation, intervention, and analysis is presented in Figure 6.

### 2.6. Statistical Analysis

Data analysis was performed using Microsoft Excel 2019 (Microsoft, Redmond, WA, USA) and SPSS Statistics 26 (IBM, Armonk, NY, USA) (2019). Descriptive statistics included the number of measurements (N), mean (M) ± standard error (SE), median, and minimum–maximum values. Normality was tested using the Kolmogorov–Smirnov test. Differences between baseline and follow-up values were analyzed with the Wilcoxon signed-rank test. Results were considered statistically significant at *p* ≤ 0.05.

Generative artificial intelligence was used to assist in the generation of English language text, logical structuring of manuscript sections, and the formatting of figures and tables. All scientific content, data, interpretations, and conclusions were provided entirely by the authors. AI (ChatGPT, GPT-5, OpenAI, San Francisco, CA, USA) was used solely to help articulate and visualize the information in a clear and coherent manner. The final manuscript was reviewed, verified, and approved by the authors to ensure scientific accuracy and integrity.

## 3. Results

### 3.1. Hematology and PRP Analysis

Analysis of hematological parameters and PRP composition revealed a statistically significant, nearly fivefold increase in white blood cell count (*p* = 0.039731), whereas the increase in red blood cells was minor and statistically insignificant. When comparing PRP composition with whole blood morphology, PRP contained 6.3 times more neutrophils and 8.7 times more monocytes, although these differences did not reach statistical significance. A 3.8-fold increase in platelet concentration was also identified in PRP compared with blood morphology (*p* = 0.002516). A summary of hematological and PRP parameters is presented in Table 3.

### 3.2. *Periodontal Parameters*

Further analysis demonstrated that the most pronounced periodontal pocket depth and horizontal bone loss occurred in the canine teeth. In the canine group, periodontal pocket depth decreased by 1.70 mm between day 0 and day 30 following PRP injections, compared with 0.89 mm in the control group (*p* < 0.05). Similarly, horizontal bone loss decreased by 1.19 mm in the PRP group and 0.94 mm in controls (*p* < 0.05). PRP injections reduced the stage of periodontitis by 1.5-fold over 30 days. Gingival index decreased threefold between day 0 and day 30 in the PRP group, and was reduced twofold compared with controls. Periodontal pocket depth was reduced twofold after PRP injections between day 0 and day 30, and 1.5-fold compared with controls. Horizontal bone loss decreased more than twofold after PRP therapy compared with baseline and was reduced 2.2-fold compared with control group (Table 4).

## 4. Discussion

This study demonstrated that administering PRP injections into gingival, submucosal, and periodontal pocket sites—without the use of additional activating agents—significantly improved treatment efficacy in dogs with stage 2–3 periodontitis. As dogs treated with PRP showed superior periodontal outcomes compared with the control group, these findings indicate that PRP therapy, even when applied without activating agents, provides measurable clinical benefits in routine veterinary dental practice. Improvements were observed over a 4-week period without complications. Across parameters including periodontitis stage, gingival index, periodontal pocket depth, and horizontal bone loss, outcomes improved almost twofold compared with conventional therapy. Although slight swelling was observed on the first day after the injections, no discomfort or inflammatory reactions were observed during the follow-up visits. Hypersalivation was observed for the first 2–3 h after the injections, but no long-term lesions or symptoms were observed later. The swelling at the injection site resolved within a maximum of 24 h. Therefore, no side effects, discomfort or pain caused by long-term injections were observed.

Our findings indicate that PRP therapy produced statistically significant improvements within 30 days, whereas in the study by Chung et al. (2023), meaningful improvement was observed only after 56 days, with no significant changes at day 28 [9]. Harnack et al. (2009) also reported in humans that PRP efficacy depends on disease stage, with diminished effects in later stages where periodontal tissues are severely compromised by bacterial and immune-mediated damage [25].

Our study confirmed that PRP injections significantly reduced periodontal pocket depth compared with conventional cleaning. Abdul Ameer et al. (2018) reported statistically significant reductions in humans after one month of PRP therapy, with an average decrease of 0.72 mm [26]. In contrast, our study achieved a greater mean reduction of 1.70 mm. PRP injections in our study reduced pocket depth by approximately 50% more than conventional dental cleaning, with the most pronounced reductions observed in premolar teeth (61.64%). Chung et al. (2022) also reported periodontal pocket depth reductions of approximately 2 mm [9]. We also observed marked improvements in parameters related to hard tissue. In our study, horizontal bone loss decreased by 2.12 mm within 30 days of PRP injections, compared with 1.4 mm over 56 days in the study by Chung et al. (2023) [9]. Although soft tissues typically regenerate faster than hard tissues, our results suggest that PRP may accelerate both processes in dogs.

Analysis of the gingival index in our study revealed a threefold reduction within 30 days in the PRP group, with statistically significant differences from controls. Similar reductions were reported by Chung et al. (2023), though their improvements were detected within 14 days, possibly due to the addition of calcium chloride activator and higher concentrations of growth factors [9]. Since we did not measure growth factor levels in this study, direct comparison with those results is limited; however, our aim was to evaluate the effectiveness of non-activated PRP as a practical tool in routine treatment of periodontitis. We acknowledge that the absence of growth factor quantification represents a limitation.

Evaluation of blood and PRP parameters revealed statistically significant increases in leukocytes and platelets, by approximately 5-fold and 3.3-fold, respectively. Comparable results have been reported by other authors: Hatakeyama et al. (2013) found a threefold increase in platelet count in PRP, Masuki et al. (2016) reported a fivefold increase in leukocytes in humans, and Chung et al. (2023) observed 4.5-fold higher platelet counts and 7.2-fold higher leukocyte counts in Beagle dogs [9,27,28]. The similarities across studies support the reliability of our findings, although differences in breed, age (3–4 years), and small body size of subjects in this study may have influenced PRP composition.

Compared with exogenously activated PRP, non-activated PRP undergoes physiologic activation at the site of injection, which likely results in a more gradual release of growth factors. Therefore, this slower release of bioactive substances may contribute to a prolonged biological effect and could explain why statistically significant improvements occurred at day 30.

Beyond regenerative effects, recent evidence also suggests that platelet concentrates may function as effective carriers of antimicrobial activity within periodontal pockets. A systematic review demonstrated that PRP, PRF, and especially i-PRF (Injectable Platelet-Rich Fibrin) possess intrinsic antibacterial properties against major periodontal pathogens such as *Porphyromonas gingivalis* and *Aggregatibacter actinomycetemcomitans*. Notably, i-PRF produced the widest zones of bacterial inhibition, followed by PRP and then PRF, indicating differential antimicrobial potency among these preparations [4]. Proposed mechanisms include the release of antimicrobial peptides, oxygen metabolites, and cooperative interactions among platelets, leukocytes, and entrapped microorganisms that enhance pathogen clearance [4]. These findings support the concept that platelet concentrates can act not only as scaffolds and reservoirs of growth factors but also as biologically active carriers capable of delivering antimicrobial molecules directly into periodontal defects. This dual function—simultaneously reducing bacterial burden and promoting tissue healing—further strengthens the rationale for their use as adjunctive therapies in periodontal disease management.

Previous research also indicates that patient factors affect PRP efficacy. Hirai et al. (2013) studied Shiba Inu dogs and found a correlation between age and the degree of periodontitis and found that the incidence of gingivitis and periodontitis was higher in middle-aged and elderly dogs than in young dogs [29]. Wallis & Holcombe (2020) found that smaller body size correlates with higher risk and severity of periodontitis [2]. de Marcos Carpio et al. (2021) also reported that PRP effectiveness decreases with age and that body weight negatively correlates with platelet concentration in PRP, implying lower platelet counts and diminished efficacy in heavier animals [30]. These considerations should be taken into account when interpreting treatment outcomes and tailoring PRP protocols in clinical veterinary practice.

## 5. Conclusions

The use of PRP administered via gingival, submucosal, and periodontal pocket injections, without additional activating agents, significantly improves treatment outcomes in small-breed dogs with stage 2–3 periodontitis. When applied in conjunction with routine dental hygiene procedures, PRP is a safe, minimally invasive, and effective adjunct therapy that enhances both soft and hard tissue regeneration.

## Figures and Tables

**Table 1:** Selection criteria for small-breed dogs included in the study.

Inclusion Criteria	Exclusion Criteria
Adult small-breed dogs	Medium and large breed dogs
3–4 years of age	Stage 1 and stage 4 periodontitis
Stage 2 or stage 3 periodontitis	Patients that have concomitant diseases
Dry food only	Patients who received home dental care
	Patients who received medications or supplements

**Table 2:** The Scoring Criteria of Gingival Index (GI)^20^.

**GI = 0**	healthy gums
**GI = 1**	slight inflammation, possible slight discoloration or edema of the gums, no spontaneous bleeding on probing.
**GI = 2**	moderate inflammation, red gums, pronounced edema, bleeding on probing.
**GI = 3**	significant inflammation, pronounced gingival redness, edema and ulceration, characterized by spontaneous bleeding

**Table 3:** Red blood cell, white blood cell and platelet median (min-max) value counts between whole blood and PRP.

	Whole Blood	PRP	*p*
Red blood cells × 10^12^/L	5.7 (5.5–7.4)	6.4 (5.9–9.6)	0.091372
White blood cells × 10^9^/L	12.4 (5.8–15.8) ^1^	61.6 (59.2–124.7) ^1^	0.039731
Neutrofils × 10^9^/L	7.7 (5.6–14.6)	48.2 (26.4–88.8)	0.682143
Monocites × 10^9^/L	0.9 (0.74–1.1)	7.8 (3.2–12.4)	0.572324
Platelets K/µL	340 (228–416) ^2^	1288 (689–1517) ^2^	0.002516

^1^ Significant difference between white blood cells in whole blood and PRP. ^2^ significant difference between platelets in whole blood and PRP. PRP—Platelet-rich plasma.

**Table 4:** The median (min–max) values of periodontal parameters of control (dental scaling alone) and PRP (dental scaling with PRP) groups.

	Stage of Periodontitis		Gingival Index		Periodontal Pocket Depth (mm)		Horizontal Bone Loss (mm)	
	Control	PRP	Control	PRP	Control	PRP	Control	PRP
**Day 0**	2 (2–3)	3 (2–3)	2 (1–3)	3 (2–3)	4 (1–7)	4 (1–8)	3.91 (1.12–5.74)	4.20 (1.18–8.54)
**Day 30**	2 (1–3)	2 (1–2)	2 (1–2) *	1 (0–2) *	3 (1–5) **	2 (1–4) **	4.65 (1.30–7.21) ***	2.08 (1.03–5.35) ***
***p***	0.025	0.000183	0.025	0.000074	0.000	0.000	0.000	0.000

* Significant difference in **gingival index** between control and PRP groups at day 30 (*p* = 0.025). ** significant difference in **periodontal pocket depth** between control and PRP groups at day 30 (*p* = 0.047). *** significant difference in **horizontal bone loss** between control and PRP groups at day 30 (*p* = 0.001). PRP—Platelet-rich plasma.

## Data Availability

The data presented in this study are available on request from the corresponding author. The data are not publicly available due to privacy or ethical restrictions.

## Abbreviations

The following abbreviations are used in this manuscript:

PRP	Platelet-Rich Plasma
PDGF	Platelet-Derived Growth Factor
FGF	Fibroblast Growth Factor
VEGF	Vascular Endothelial Growth Factor
EGF	Epidermal Growth Factor
PRF	Platelet-Rich Fibrin
PRGF	Plasma Rich In Growth Factors
CEJ	Cemento–Enamel Junction
ACP	Autologous Conditioned Plasma
i-PRF	Injectable Platelet-Rich Fibrin
